# Acetylation Reader Proteins: Linking Acetylation Signaling to Genome Maintenance and Cancer

**DOI:** 10.1371/journal.pgen.1006272

**Published:** 2016-09-15

**Authors:** Fade Gong, Li-Ya Chiu, Kyle M. Miller

**Affiliations:** Department of Molecular Biosciences, Institute for Cellular and Molecular Biology, The University of Texas at Austin, Austin, Texas, United States of America; National Institute of Environmental Health Sciences, UNITED STATES

## Abstract

Chromatin-based DNA damage response (DDR) pathways are fundamental for preventing genome and epigenome instability, which are prevalent in cancer. Histone acetyltransferases (HATs) and histone deacetylases (HDACs) catalyze the addition and removal of acetyl groups on lysine residues, a post-translational modification important for the DDR. Acetylation can alter chromatin structure as well as function by providing binding signals for reader proteins containing acetyl-lysine recognition domains, including the bromodomain (BRD). Acetylation dynamics occur upon DNA damage in part to regulate chromatin and BRD protein interactions that mediate key DDR activities. In cancer, DDR and acetylation pathways are often mutated or abnormally expressed. DNA damaging agents and drugs targeting epigenetic regulators, including HATs, HDACs, and BRD proteins, are used or are being developed to treat cancer. Here, we discuss how histone acetylation pathways, with a focus on acetylation reader proteins, promote genome stability and the DDR. We analyze how acetylation signaling impacts the DDR in the context of cancer and its treatments. Understanding the relationship between epigenetic regulators, the DDR, and chromatin is integral for obtaining a mechanistic understanding of genome and epigenome maintenance pathways, information that can be leveraged for targeting acetylation signaling, and/or the DDR to treat diseases, including cancer.

## Introduction

Genome maintenance relies on the precise replication and repair of our genetic information. This is a daunting task given the 3 X 10^9^ bp size of the human genome and the presence of thousands of DNA lesions that are generated per cell per genome every day by various genotoxic processes and agents. To maintain genome integrity in these adverse conditions, cells contain DNA damage response (DDR) pathways that detect, signal, and repair DNA lesions [1,2]. Multiple DNA repair pathways are present to accommodate diverse DNA lesions. A particularly cytotoxic DNA lesion is the DNA double-strand break (DSB). DSBs can promote mutations, DNA degradation, or ligation to other DNA ends, resulting in genome instability. DSBs are repaired mainly by non-homologous end-joining (NHEJ) and homologous recombination (HR) in mammalian cells [3]. NHEJ ligates the two broken DNA ends together [4], whereas HR uses a homologous DNA template to accurately copy and repair the DSB [5,6]. The importance of DDR pathways is highlighted by the various diseases associated with DDR defects, including neurodegenerative disorders, immune deficiencies, and cancer [1,7].

Eukaryotic nuclear DNA is organized into chromatin [8]. The basic unit of chromatin is the nucleosome, consisting of DNA wrapped around histone proteins [8,9]. Chromatin organizes the genome and controls its accessibility, making chromatin integral for DNA-based processes. Chromatin is highly modified by posttranslational modifications (PTMs), including phosphorylation, methylation, and acetylation [10–13]. PTMs regulate chromatin structure as well as modulate chromatin interactions of “reader” proteins that contain PTM binding domains [11,14–16]. Epigenetic changes and mutations within chromatin regulatory proteins are observed in different diseases, including cancer [17]. As many DDR activities occur within chromatin, understanding the interplay between chromatin and the DDR is fundamental for obtaining mechanistic details of DDR activities in both normal and diseased contexts.

Chromatin PTMs are dynamically regulated in response to DNA damage both locally at the lesion site and globally where they perform several functions [18–22]. These include modulating chromatin structure at DNA damage sites and across the genome to facilitate the DDR, including signaling [22–24], repair [25], and transcriptional responses [26,27]. Another fundamental role of PTMs is to provide docking sites for the recognition and accumulation of DDR factors at damage sites where they orchestrate DDR functions. For example, DNA damage-induced phosphorylation of histone variant H2AX (γH2AX) is recognized by the BRCT domains of MDC1, which mediate the recruitment of downstream signaling and repair proteins to damage sites [28]. 53BP1 represents another histone PTM DDR factor reader [29,30], which recognizes the bivalent marks H4K20me2 and H2AK13/15ub at damage sites [31,32]. Histone PTMs and their DDR functions have been comprehensively reviewed [18–21,33]. Here, we discuss acetylation signaling with a focus on acetylation readers that are involved in mammalian genome maintenance and DNA repair. We also consider DDR-related acetylation signaling in cancer and its potential impact on cancer therapies.

## Acetylation Signaling in the DDR

**Fig 1:**
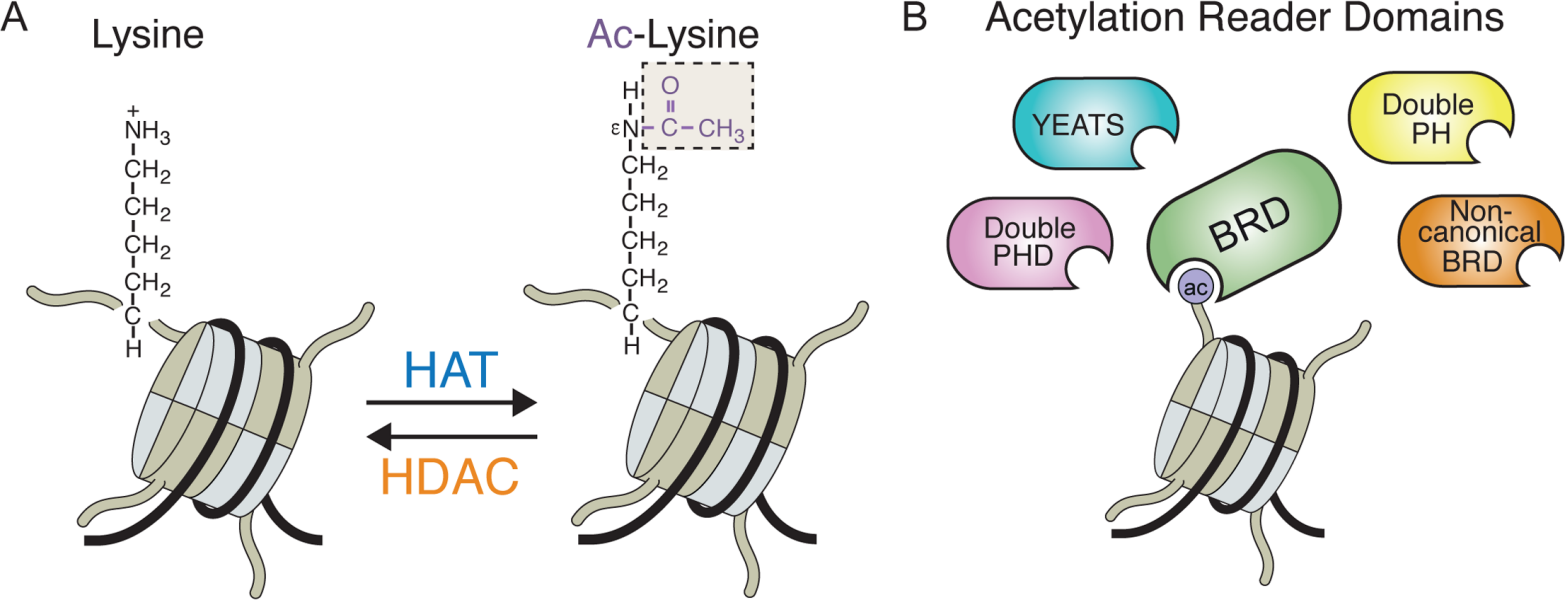
Acetylation signaling. **(A)** HATs add an acetyl (Ac) moiety on the ε-amino group of a lysine residue, and HDACs reverse this reaction. **(B)** Acetylated lysines on histones are recognized and bound by proteins containing BRD domains and other acetyl-lysine binding domains. Abbreviations: ac, acetylation; HAT, histone acetyltransferase; HDAC, histone deacetylase; BRD, bromodomain; YEATS, Yaf9, ENL, AF9, Taf14, and Sas5; PHD, plant homeodomain; PH, pleckstrin-homology.

Acetylation is the covalent attachment of an acetyl-group (-COCH3) to the ε-amino groups of a lysine residue on histones and non-histone proteins by histone acetyltransferases (HATs) [34,35], which can be removed by histone deacetylases (HDACs) [36] (Fig 1A). Acetylation levels are regulated by the concerted activities of HATs and HDACs [21,35,36]. The importance of acetylation signaling is well established in many cellular processes, including transcription and the DDR [14]. One salient function of acetylation is to regulate chromatin structure. This is exemplified by H4K16ac, which blocks inter- and intra-chromosomal folding to promote an open chromatin structure [37]. Acetylated proteins are also bound by acetyl-lysine reader proteins. The principal acetylation recognition proteins contain the acetyl-lysine binding bromodomain (BRD), although other domains have acetyl-lysine interaction capabilities (Fig 1B) [16,38,39]. Since acetylation changes upon UV damage were observed over 30 years ago, numerous DNA damage responsive acetylations have been reported [21,40]. The involvement of acetylation reader proteins in deciphering these signals to promote the DDR is less clear. Recent studies have identified over one-third of human BRD proteins directly responding to DNA damage, suggesting that, collectively with HATs and HDACs, the entire acetylation signaling machinery orchestrates DDR activities within chromatin (Table 1) [41–44].

**Table 1:** DDR functions of mammalian acetylation readers.

Domain	Protein	Complex	Histone Binding Ac targets	Damage recruitment	DDR Ac binding	DDR functions	Reference
**BRD**	ATAD2	-	K3K14, H4K5	-	-	-	[181,182]
	ATAD2B	-	H4K5	-	-	-	[38]
	ACF1 (BAZ1A)	CHRAC, ACF	-	Laser, R.E., UV	N	NHEJ, HR, NER, Checkpoint	[41,94,95,98]
	WSTF (BAZ1B)	WICH, B-WICH	-	Laser, UV	N	NER, DDR signaling, Checkpoint	[41,95,97,98]
	BAZ2B	-	H3K14	-	-	-	[38]
	BPTF	NURF	H4K5/16	Laser	-	-	[41]
	BRD2	-	H3K14, H4K5/8/12/16/20	-	-	-	[55]
	BRD3	-	H3K14/18, H4K5/8/12/16/20	-	-	-	[55]
	BRD4	-	H3K9/14, H4K5/8/12/16	-	Y	Regulate chromatin	[55,71]
	BRD7	-	H3K9/14, H4K8/12/16	-	-	Regulate p53 activity	[91,92,183]
	BRDT	-	H2BK12/15, H3K9/14/18/23, H4K5/8/12/16	-	-	-	[55]
	BRPF1	-	H2AK5, H3K14, H4K12	-	-	-	[184]
	CECR2	CERF	H3K9/14	-	Y	DDR signaling	[38,185]
	CBP (CREBBP)	-	H2BK85, H3K9/14/36/56, H4K12/20/44	Laser, R.E.	-	NHEJ, HR, NER	[44,55,76,186,187]
	GCN5	SAGA, ATAC	H2AK5, H3K9/14, H4K8/14/16	Laser, UV	-	NER, Regulate chromatin	[27,41,55,77,83]
	BAF180 (PBRM1)	PBAF	H3K4/9/14/18/23	Laser	Y	Transcription repression, Regulate p53 activity, Promote cohesion	[42,55,89,91]
	p300	-	H3K36/56, H4K12/20/44	Laser, R.E., UV	-	NHEJ, HR, NER, DDR signaling, Checkpoint, Regulate p53 activity	[41,44,55,76,176,186–192]
	PCAF	ATAC	H3K9/14/36, H4K8/16/20	Laser	-	Regulate p53 activity	[41,55,78,191]
	BRM (SMARCA2)	BAF	H3K9/14, H4K5/8/12/16	Laser	Y	NHEJ, DDR signaling	[41,44,55,193]
	BRG1 (SMARCA4)	BAF, PBAF	H2BK5, H3K9/14, H4K8/K12/K16	Laser, UV	Y	NER, Regulate chromatin, Transcription repression, Checkpoint, DDR signaling	[41,42,44,55,85–87,193,194]
	TAF1	-	H3K9/14, H4K5/8/12/16	-	-	Checkpoint	[195]
	TRIM24	-	H3K23, H4K16	Laser	-	Regulate p53 activity	[41,69,196]
	KAP1 (TRIM28)	-	No binding	IR, Laser	N	DDR signaling, Heterochromatin repair, Transcription repression	[64,66,68,113,197]
	TRIM33	-	H3K18/23	Laser	-	Regulate ALC1 activity	[41,43,70]
	ZMYND8	NuRD	H4K5/8/12/16	Laser	Y	Transcription repression, HR	[41]
	ZMYND11	-	No binding	-	-	-	[160]
	Uncharacterized acetylation interactions: ASH1L, BAZ2A, BRD1, BRD8, BRD9, BRPF3, BRWD1, BRWD3, MLL, PHIP, SP100, SP110, SP140, SP140L, TAF1L, TRIM66						
**Double PHD**	DPF3	PBAF	H3K14	-	-	-	[114]
	MORF	-	H3K9/14	-	-	-	[115]
	MOZ	-	H3K9/14	-	-	-	[115,116]
**YEATS**	AF9	-	H3K9/18/27	-	-	Transcription repression	[39,119,120]
	ENL	-	H3K9/27	-	-	Transcription repression, NHEJ	[119,120]
	GAS41	NuA4, SRCAP	H3K9/27	-	-	-	[119]
	Uncharacterized acetylation interactions: YETS2						
**Non-canonical BRD**	DNA-PKcs	-	H2AXK5	IR, Laser	Y	DDR signaling, NHEJ, Transcription repression	[112,122,198]

Acetylation readers and their histone targets are provided. The reported damage recruitment, acetylation binding in the DDR, and DDR functions of acetylation readers are listed. Abbreviations: DDR, DNA damage response; NHEJ, nonhomologous end-joining; HR, homologous recombination; NER, nucleotide excision repair; IR, ionizing radiation; R.E., restriction enzyme; CHRAC, chromatin accessibility complex; WICH, WSTF-ISWI chromatin remodeling complex; NURF, nucleosome-remodeling factor; CERF, CECR2-containing remodeling factor; SAGA, Spt-Ada-Gcn5-acetyltransferase; ATAC, Ada2-containing chromatin-modifying complex; BAF, BRG1- or HBRM-associated factor; PBAF, polybromo-associated BAF; NuRD, nucleosome remodeling and histone deacetylase complex; NuA4, nucleosome acetyltransferase of H4 (or TIP60-p400 complex); SRCAP, Snf-2-related CREB-binding protein activator protein.

## HATs, HDACs, and the DDR

The involvement of mammalian HATs and HDACs in the DDR is exemplified by their common localization to damage sites [21]. The HAT TIP60 (KAT5), a key component of the NuA4 complex, is involved in DSB repair from early signaling events to downstream repair pathway choices [45]. Upon DNA damage, TIP60 acetylates histones and the Ataxia telangiectasia mutated (ATM) kinase to enhance its activity [46–48]. Acetylated H4K16 by TIP60 promotes HR repair, whereas deacetylation facilitates NHEJ [47,49]. The DSB pathway choice regulated by TIP60 occurs in part by its ability to acetylate H2AK15, which impedes RNF168 ubiquitylation to block 53BP1 recruitment, thereby promoting HR [48]. The HAT Males absent on the first (MOF) regulates global H4K16Ac levels, and its loss impacts the DDR. Indeed, cells deficient of MOF exhibit impaired recruitment of DDR factors and a reduced capacity for DSB repair by HR and NHEJ [50–52]. Among HDACs, HDAC1 and HDAC2 play particularly important roles in DSB repair by deacetylating histones, including H3K56ac and H4K16ac [27,49]. HDACs can also target both histone and non-histone proteins. For example, SIRT6 deacetylates H3K56 and also the DDR factor CtIP, which regulates both the rapid recruitment of the remodeling factor SNF2H and the activity of CtIP at damage sites to promote DNA repair [53,54]. These studies highlight important functions of HATs and HDACs in the DDR (reviewed in [21]).

## Readers of Acetylated Lysines in the DDR: Bromodomain Proteins

Emerging evidence has highlighted the importance of acetylation readers, including BRD proteins, in the DDR. One or more BRDs are encoded in 42 human proteins [38,55]. BRD domains consist of several α-helices linked by loops that form a hydrophobic cavity that specifically recognizes acetyl-lysines. BRD proteins can be broadly classified as HATs, components of ATP-dependent chromatin remodeling complexes, and/or transcriptional regulators [38,41,56]. Dysfunction of BRD proteins has been identified in diseases, including cancer [57]. The demonstrated druggability of the BRD by small molecule inhibitors has motivated targeting BRD proteins as cancer therapies [58–61].

### Regulation of chromatin states by BRD proteins in the DDR

Chromatin exists between open and condensed states that impact its functionality. In heterochromatin, DSBs require specific factors as well as chromatin remodeling complexes to overcome the chromatin barrier to allow damage recognition and signaling to promote repair [62,63]. The BRD protein KAP1 (TRIM28) functions in DSB repair within heterochromatin. ATM phosphorylates KAP1 in response to DSBs [64], which disperses the nucleosome remodeler CHD3 from chromatin to trigger chromatin relaxation, allowing repair to occur within heterochromatin [64–66]. Three KAP1 tripartite motif-containing (TRIM) family paralogs (TRIM24, TRIM33, and TRIM66) also contain BRDs [67]. TRIM24 and TRIM33 are recruited to DNA damage and are involved in the DDR [41,43]. Although KAP1 BRD lacks acetyl-lysine binding [68], the plant homeodomain (PHD)-BRD tandem domains of TRIM24 and TRIM33 can read methylated or acetylated histones [69,70]. Whether these TRIM BRD proteins act interdependently or independently in the DDR awaits further investigation.

An isoform of BRD4, a member of the bromodomain and extra-terminal (BET) family of BRD proteins, has been reported to insulate chromatin to modulate γH2AX spreading around damaged DNA [71]. BRD4 contains two BRD domains and functions as a regulator of various transcriptional processes [72]. Successful targeting of the BRDs of BRD4 by small molecule inhibitors has gained widespread attention for its potential as an anticancer therapy and as a proof of concept for the “druggability” of BRD proteins [58,59]. In the DDR, inhibition of BRD4 with the BRD targeting inhibitor JQ1 resulted in increased DNA damage signaling of γH2AX formation, while survival from DNA damage was improved [71]. These data implicate the BRD of BRD4 as a key domain involved in regulating the cellular response to DNA damage. The BRDs of BRD4 have been shown to bind acetylated histone H3 and H4 [55]. BRD4 has also been reported to act as a HAT, acetylating H3 and H4 [73]. It is unclear how BRD4 recognizes damaged chromatin and if its HAT activity participates in its DDR functions. It will be important to distinguish the specific acetylated residues read and/or acetylated by BRD4 in cells upon DNA damage compared to those targeted during normal transcription to understand mechanistically how BRD4 promotes both transcriptional regulation in unperturbed cells and DNA damage signaling in response to DNA damage.

### BRD-containing HATs in the DDR

The BRD-containing HATs GCN5, PCAF, p300, and CBP exist in multiple complexes from yeast to human that regulate transcription both through their enzymatic HAT activity and also through interacting with chromatin [74,75]. The BRD in HATs can broadly recognize acetyl-lysines mainly on H3 and H4 tails but also on H2A/H2B and non-histone factors [55]. For example, GCN5 and PCAF primarily acetylate H3 tails, but their BRDs bind H3ac and H4ac. This suggests the BRD may promote HAT activity by binding certain acetylated residues that would then promote the acetylation of other targets. Interestingly, these HATs are all recruited to DNA damage, where they participate in various aspects of the DDR including NER, DSB repair, and checkpoint regulation [21,41,44,76–78]. The DDR function of BRDs within these HATs is uncharacterized. Future studies identifying both HAT targets and BRD recognition signals will be essential to understand how HATs coordinate their catalytic activities with BRD reader capabilities to promote the DDR.

### BRD protein components of chromatin remodeling complexes in the DDR

**Fig 2:**
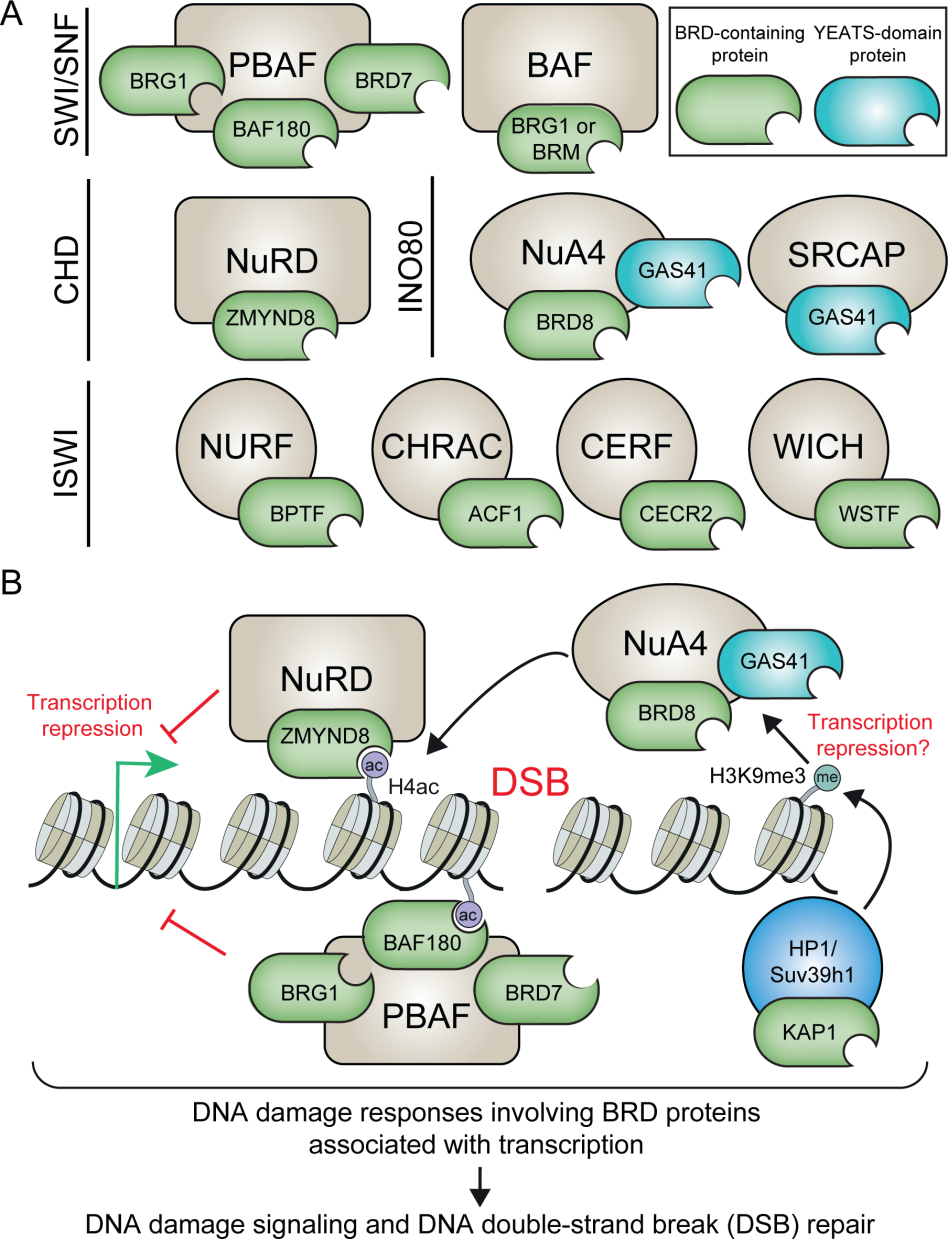
**Acety-lysine readers within (A) chromatin remodeling complexes and (B) BRD protein pathways involving DNA damage within transcriptionally active chromatin. (A and B).** Abbreviations: BRD, bromodomain; DSB, DNA double-strand break; ac, acetylation; me, methylation. Common and alternative names include: BRG1 (SMARCA4), BRM (SMARCA2), BAF180 (PBRM1), ACF1 (BAZ1A), WSTF (BAZ1B), KAP1 (TRIM28).

ATP-dependent chromatin remodeling complexes control the accessibility of chromatin factors to DNA by disrupting DNA–histone interaction, sliding/evicting nucleosome, or altering nucleosome composition, thereby regulating chromatin-based processes including transcription and DNA repair [79,80]. Chromatin remodeling complexes are organized into four principal families: switching defective/sucrose nonfermenting (SWI/SNF), imitation switch (ISWI), chromodomain, helicase, DNA binding (CHD), and inositol requiring 80 (INO80) [79]. In mammalian cells, ten BRD proteins have been identified within these four chromatin remodeling families (Fig 2A) [38,41,79]. Acetylation reader activities appear to represent a universal component of these complexes, and several studies are now defining their DDR functions within chromatin remodeling complexes.

The mammalian SWI/SNF complexes BAF and PBAF contain BRD proteins including BRG1 (SMARCA4) and BRM (SMARCA2) that are involved in the DDR [81]. The ATPase subunit BRG1 functions in both complexes, whereas the ATPase BRM is exclusive to BAF. BRG1 is recruited to DNA damage and functions in the DDR, including through the binding of DSB-associated nucleosomes containing γH2AX, which requires the BRD to recognize GCN5-dependent H3ac [82–87]. BRM associates with DNA damage in a p300/CBP-dependent manner, which facilitates the recruitment of KU70 for NHEJ [41,44]. The role of BRM BRD in the DDR is unknown. PBAF contains two additional BRD proteins: BAF180 (PBRM1) and BRD7. BAF180 encodes six BRDs and is linked to the DDR [42,88,89]. With BRG1, BAF180 regulates PBAF in damage-induced transcription repression (see below) [42]. BAF180 also promotes cohesion, a function impaired upon mutations within its two BRDs, which leads to genome instability [89]. BRD7 interacts with BRCA1 and regulates p53, suggesting its involvement in the DDR [90–92].

The chromatin remodeler ISWI exists in many complexes that contain multiple BRD proteins [93]. The BRD protein ACF1 (BAZ1A) is a non-catalytic subunit of CHRAC and ACF ISWI complexes. ACF1 is recruited to damage, where it helps recruit KU for NHEJ [94]. ACF1 also regulates the G2/M DNA damage checkpoint [95]. Although ACF1 damage-recruitment is independent from its BRD, its chromatin binding domains could still promote DDR functions. WSTF (BAZ1B) is specific for the WICH ISWI complex [96]. WSTF is recruited to DNA damage where it regulates γH2AX as a kinase that targets H2AX Y142 [41,95,97]. The potential interplay between remodeling, acetylation binding, and kinase activity of this BRD DDR factor is worth investigating. ACF1 and WSTF are also recruited to UV-C laser irradiation and function in NER [98]. Currently, acetylation targets of ACF1 or WSTF BRDs remain unidentified, making mechanistic interpretations of these results challenging. The ISWI complex NURF also contains Bromodomain PHD Finger Transcription Factor (BPTF) that is recruited to DNA damage [41]. The BRD of BPTF recognizes H4K16ac to regulate transcription [99]. It will be interesting to determine whether BPTF participates in an acetylation-dependent DDR activity involving H4K16ac.

The CHD member nucleosome remodeling and histone deacetylase (NuRD) plays well-established roles in the DDR [100–103]. Although the canonical NuRD complex lacks any BRD protein, multiple studies have identified BRD proteins associated with this complex at DNA damage sites. For example, ZNF827 can specifically recruit NuRD to ALT telomere to regulate HR [104], whereas the BRD protein ZMYND8 recruits NuRD to DSBs within actively transcribing chromatin [41]. The INO80 chromatin remodeling family includes the NuA4 complex, which displays various DDR functions [45,63,79]. In addition to remodeling activity by p400, NuA4 also acetylates histones through its associated HAT activity by TIP60. Both chromatin remodeling and HAT activities play critical roles in DNA damage signaling and repair, including promoting HR and suppressing alternative-NHEJ [47,105–109]. The NuA4 complex contains the BRD protein BRD8 as well as GAS41, a YEATS protein with acetyl-lysine recognition capabilities [79,110]. These acetylation readers have the potential to coordinate the DDR functions of NuA4, although further studies are needed to elucidate the mechanistic details of how these large multi-subunit complexes promote the DDR in the context of acetylation. Thus, these studies highlight the involvement of BRD proteins within chromatin remodelers that have the potential to link acetylation signaling with the DDR.

### Transcription, DNA damage, and BRD proteins

Transcription can be hindered by DNA damage, requiring molecular retooling of the chromatin environment to avoid conflicts between DNA repair and active transcription. Indeed, transcriptionally active genes located nearby DSBs are repressed by the DDR kinases ATM [111] and DNA-PK [112] to facilitate DSB repair. Acetylation readers have been identified that coordinate the DDR within transcriptionally active chromatin. The chromatin remodeling PBAF complex (SWI/SNF-B), which contains two BRD proteins BAF180 and BRG1, silences transcription upon DSBs and promotes NHEJ [42]. Although acetylation targets for the complex were not identified, point mutations identified in cancer genomes in BRDs of BAF180 rendered this complex defective for transcription silencing. The BRD protein ZMYND8 plays key roles in damage-induced transcriptional repression [41]. Upon DNA damage specifically within actively transcribing chromatin, ZMYND8 is recruited through its BRD to TIP60-mediated H4 acetylations. ZMYND8 associates with the NuRD complex and facilitates its accumulation at damage sites to mediate transcriptional repression and promote HR repair [41]. KAP1 participates in damage-induced formation of repressive chromatin. KAP1 is found in a methyltransferase complex with HP1 and Suv39h1, which promote H3K9me3 upon DSBs to form repressive chromatin transiently at break sites [113]. How KAP1-dependent H3K9me3 affects transcription within these damaged regions is unclear. These examples provide key examples for how BRD proteins participate in transcription responses to DNA damage (Fig 2B). Whether these pathways are coordinated or act independently at DSBs across varied chromatin landscapes needs further investigation. Interestingly, NuRD and KAP1 complexes are associated with gene repression, whereas PBAF is associated with active transcription. Identifying DDR-specific regulatory cues that switch these transcriptional regulators into DNA damage factors is needed to create a full view of the interplay between transcription and the DDR. It seems clear that transcription-associated DDR complexes must perform their normal functions in gene regulation while also being able to promote DNA repair of damaged DNA within transcriptionally active chromatin.

## Non-BRD Acetylation Binding Proteins in the DDR

Several domains in addition to the BRD can bind acetylated lysines, including double PHD fingers [114], tandem PHD fingers [115,116], double pleckstrin homology (PH) domain [117], and the YEATS domain [39]. Four YEATS-containing proteins have been identified in humans and their functions in various cellular processes identified [118]. Several YEATS proteins participate in the DDR. In yeast, the YEATS domain of Taf14 binds H3K9ac, a mark that is responsive to DNA damage in human cells [27]. Disruption of Taf14 acetylation interactions impairs the DDR and sensitizes cells to DNA damaging agents [119]. The human YEATS protein ENL, a transcriptional elongation factor, is phosphorylated by ATM in response to DSBs. Phosphorylated ENL interacts with Polycomb Repressive Complex 1 specifically at DSBs to promote lesion-induced transcriptional repression [120]. Loss of ENL and transcriptional repression at DSB sites reduces the association of the NHEJ factor KU with damage sites, suggesting that this pathway acts to repress transcription in the presence of a DSB to allow its repair. Yeast Yaf9 and its close human homolog GAS41 are conserved components of the NuA4, a complex involved in the DDR (Fig 2) [105,107,118,121]. The DNA-dependent protein kinase, catalytic subunit, DNA-PKcs, contains a bromodomain-like module that recognizes TIP60-dependent H2AX K5-Ac to promote the formation of γH2AX at DSBs [122,123]. Thus, acetylation readers in addition to BRD proteins are also critical facilitators of the DDR.

## Cancer Epigenetics: Acetylation Signaling

### Acetylation modifiers and readers in cancer

**Fig 3:**
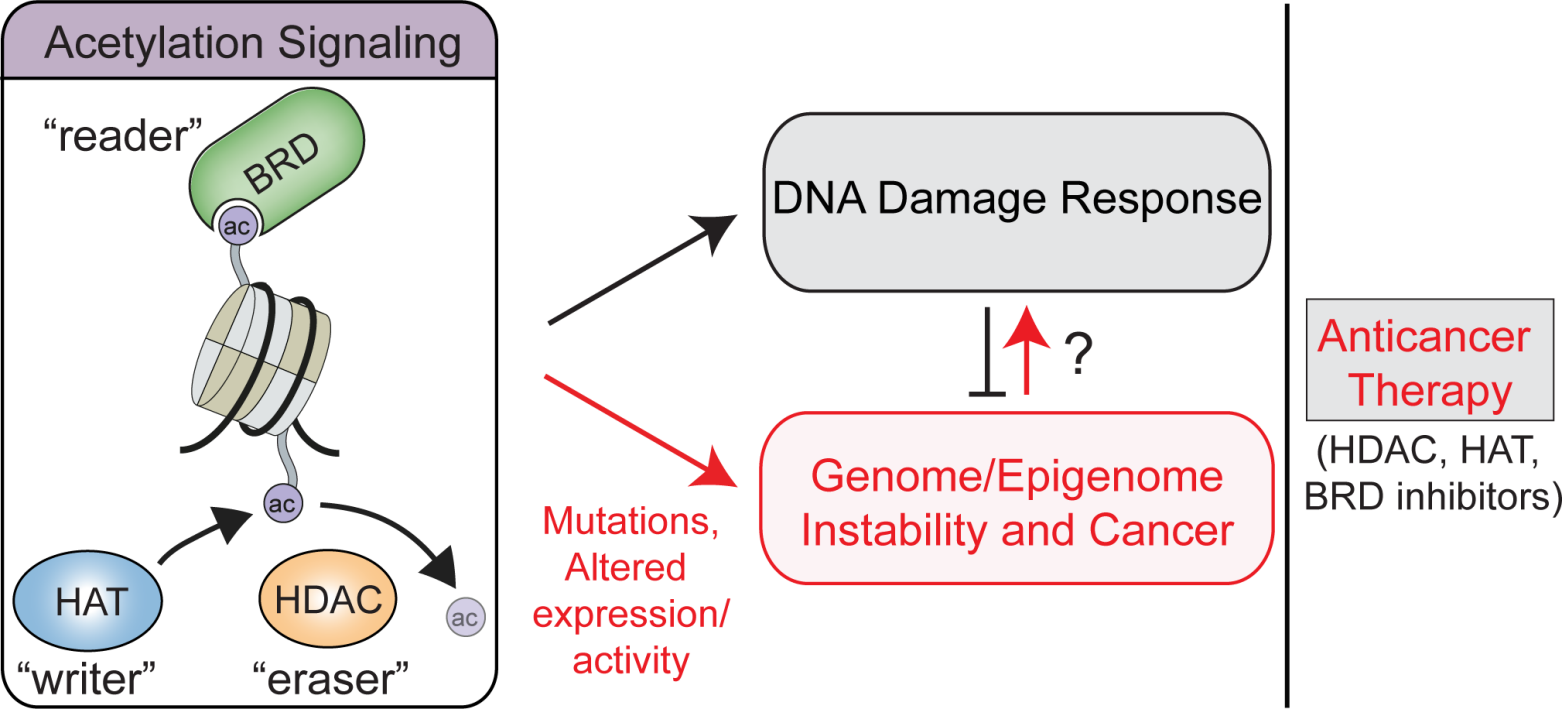
Model for the contribution of acetylation signaling in the DDR, cancer, and anticancer therapies that target epigenetic acetylation pathways. Abbreviations: ac, acetylation; BRD, bromodomain; HAT, histone acetyltransferase; HDAC, histone deacetylase.

Changes in acetylation signaling resulting from misregulated HATs or HDACs can cause abnormal gene expression patterns, including activation of proto-oncogenes and silencing of tumor suppressor genes [74,124,125] as well as impair DNA damage responses [21], which collectively can impact genome–epigenome stability (Fig 3). Altered acetylation signaling pathways have been identified in numerous cancers. Various genetic alterations in HATs have been found in hematological and solid cancers [124]. HATs can exhibit altered substrate targeting through mislocalization, aberrant protein interactions, or remodeled activities that can disrupt normal cell function. For instance, inactivating or truncated mutations in CBP/p300 HAT catalytic domains, which impair acetylation of H3K18 and non-histone substrates BCL6 and p53, have been identified in cancers [126–128]. BCL6 and p53 are transcriptional regulators involved in the DDR, and decreased acetylated BCL6 and p53 may enhance DNA damage tolerances, which can favor cancer survival [129–131]. Of note, no significant gene expression differences were found in mutant versus wt CBP/p300 containing small cell lung cancers, suggesting that mechanisms other than altered gene expression may contribute to these cancer-associated HAT mutations [128]. Given the broad involvement of CBP/p300 in gene regulation and the DDR, it remains unclear the primary cellular targets of these HATs in cancer. Analysis of genome stability pathways and acetylation reader associations with chromatin in CBP/p300 mutant cancers could provide answers to these outstanding questions.

Decreased histone acetylation levels are most frequently observed in cancer [132], which, in addition to defective HAT activity, can also be a consequence of hyper-active HDACs. Altered HDAC levels, activities, and recruitment occur in cancer and all provide potential mechanisms to facilitate tumorigenesis [125,133]. For example, HDAC1 and HDAC2 expression is often enhanced in many cancers and has been correlated with transcriptional repression of tumor suppressors, including p21 [134,135]. Global reductions of H4K16ac have been identified in various cancer cell lines and tumors as being associated with tumor progression [136]. Because HDAC1 and HDAC2 deacetylate H4K16 to promote DSB repair [49], it is tempting to speculate that the observed HDAC1/HDAC2 overexpression and hypoacetylated H4K16 in cancers could deregulate the DDR, resulting in genetic and epigenetic instability that would promote tumorigenesis [137,138]. Although many HATs and HDACs are altered in cancer and concomitant histone modification changes are observed, mechanistic studies are needed to provide molecular insights into the relationship between epigenetic changes and the DDR that are involved in cancer.

Mutated or misregulated readers of acetylation, including BRD proteins, are involved in several different cancer types. The Mixed Lineage Leukemia (MLL) protein, a methyltransferase containing a BRD, is commonly fused with other chromatin proteins in haematological cancers and is able to induce leukemic transformation [139]. A non-MLL BRD contained within the MLL fusion or the loss of the BRD of MLL, a common event in MLL onco-fusion proteins, may also contribute to leukemogenesis [140–142]. For example, domain-swapping experiments identified a specific function of the CBP BRD in MLL–CBP-induced acute myeloid leukemia (AML) [143]. Recently, the development of inhibitors that block BRD acetyl-lysine interactions has uncovered the role of BRDs in several cancers [17,72]. In nuclear protein of the testis (NUT) midline carcinoma (NMC), BRD4 can be fused with the NUT protein [144,145], and these fusions are tethered to acetylated chromatin by BRDs. Inhibition of BRD-NUT by small molecule BET BRD inhibitors promoted differentiation and decreased MYC expression to inhibit proliferation [58,146]. In leukemia with MLL fusions, BRD4 can interact with MLL fusions and associated proteins, including the super elongation complex (SEC) and the polymerase-associated factor complex (PAFc), two critical regulators of transcriptional elongation [59]. Blocking BRD4 chromatin recruitment can constrain MYC and the expression of its target genes to inhibit proliferation and promote apoptosis [59,147–149]. TRIM BRD proteins have been linked to cancer [67], and the chromatin binding module including the BRD of TRIM24 has been implicated in transcription regulation of cancer-specific genes in breast [69] and prostate cancers [150]. Thus, BRD proteins are involved in several cancers, but whether their DDR activities also play a role in tumorigenesis remains an open question.

Components of several chromatin-remodeling complexes are highly mutated in cancer, including BRG1 and BAF180 in SWI/SNF and CHD4 in NuRD [110,151]. Mutations within these complexes have been associated with aberrant gene expression and genome instability [152,153], although it is not fully understood if DDR functions of these mutated complexes impact cancer. The BRD protein ZMYND8 is a component of the NuRD complex that, along with CHD4, displays mutations or altered expression in various cancers [154–158]. Aberrant regulation of the ZMYND8-NuRD DDR pathway could disrupt its transcription and DNA repair functions, resulting in genome instability [41]. In addition, enhancer-bound ZMYND8 has been shown to act as a controller for expression of enhancer RNA (eRNA), which are short, non-coding RNAs transcribed from enhancers [159]. Loss of ZMYND8 resulted in increased eRNA expression and hyper-enhancer activity that promoted cancer phenotypes [158]. The BRD protein ZMYND11, a paralog of ZMYND8, is an H3.3K36me3 reader that represses gene expression by regulating transcriptional elongation [160] and mRNA splicing [161]. Although ZMYND11 DDR functions are unknown, it appears to play a central role in cancer suppression. Indeed, ZMYND11 depletion caused up-regulation of MYC, and enhanced proliferation in cancer cells and mutations of ZMYND11 were identified in several cancers [160]. Interestingly, some mutations in the histone H3.3 variant, including at K36, have been identified in cancer, including chondroblastomas, in which high levels of mutations in H3.3 K36 and defects in HR repair are observed [162]. It is tempting to speculate that the tumor suppression of ZMYND11 could be linked to the DDR. Additional work is needed to understand the relationship between H3K36 methylation, the DDR, and cancer, information that could be clinically relevant for drugs targeting these pathways [163].

These studies highlight the involvement of acetylation “writers,” “erasers,” and “readers” in cancer. Although regulation of gene expression is most commonly associated with acetylation signaling, these pathways also play crucial roles in the DDR that promote genome and epigenome maintenance (Fig 3). How alterations in these pathways relate to DDR defects and their contributions to tumorigenesis is poorly understood. Defective DNA repair pathways are prevalent in many cancers, and genome instability is considered a hallmark of cancer. Although defects in acetylation signaling pathways could promote mutations and genome instability, these dysfunctional pathways might also provide opportunities to treat these cancers with epigenetic drugs, targeting acetylation signaling pathways as well as therapies that target cancer cells with defective DDR capacities (Fig 3). For example, PARP inhibitors can cause synthetic lethality to kill HR-defective tumors (i.e., BRCA1 and BRCA2 mutant cancers). Cancers exhibiting impaired acetylation signaling pathways resulting in defective DNA repair could be similarly treated. Understanding how DDR pathways are deregulated in acetylation signaling defective cancers can provide insights into the development and use of therapeutic strategies targeting the DDR [164].

### Targeting acetylation signaling for cancer therapy

Deregulation of acetylation signaling in cancer has been intensively investigated and led to the development of a wide variety of small molecule inhibitors targeting acetylation signaling pathways (Table 2). The recent emergence of the importance of HATs, HDACs, and BRD proteins as key mediators of the DDR and genome maintenance (Table 1) requires an understanding of how these small molecule inhibitors impact the genome integrity functions of these pathways [21]. This information can reveal new insights into their drug mechanisms, which could provide a framework for considering opportunities for combinatorial treatment using these inhibitors with traditional cancer therapies. So far, HDAC inhibitors are one of the most well-characterized epigenome-targeting drugs and show promising therapeutic efficacy toward some cancers. HDAC inhibitors are known in some cases to directly modulate the cancer epigenome, leading to changes in gene expression profiles, an effect that is proposed to promote cell cycle arrest and cell death. HDAC inhibitors can also suppress DNA damage repair capacity in cancer cells. HDAC inhibitors reduced the expression level of key repair proteins such as KU proteins in NHEJ and RAD50 in HR in various cancer cell lines [165–169]. HDAC inhibitors also elevated reactive oxygen species (ROS) levels, a potential source for DNA damage [170,171]. Because HDACs regulate the DDR, including HDAC1/2, inhibition of these HDACs resulted in hyperacetyled H4K16 and H3K56 along with defective NHEJ, thereby impairing the DDR [49]. Thus, HDAC inhibitors impair the DDR in several ways, causing sensitization of cancer cells to DNA damaging agents including radiation (Table 2) [165–167]. HAT inhibitors including curcumin [172,173] also sensitize cells to DNA damaging agents (Table 2). However, in contrast to HDAC inhibitors, the progress of HAT inhibitors in cancer treatment has been slower due to their pleiotropic effects and poor bioavailability [174]. The potent and selective HAT inhibitor C646 has displayed antitumor efficacy in several cancer types and inhibition of DNA repair (Table 2) [175,176]. Additional studies identifying the anticancer drug mechanism of these acetylation signaling inhibitors are important to aid in the further development of acetylation signaling inhibitors for the treatment of cancer.

**Table 2:** Effects on DDR by small molecule inhibitors targeting acetylation signaling.

Family	Target	Inhibitors	Phase	Tested cancer type	Effects on the DDR	References
**HAT**	CBP/p300	Curcumin	Clinical	multiple	PARPi, CPT and HU sensitivity; (-) BRCA1 mRNA; (-) ATR activity; (-) Ku70/Ku80 recruitment	[172]
	p300	C646	Preclinical	melanoma	Cisplatin sensitivity; (-) DNA repair genes mRNA i.e., Rad51; (-) γH2AX	[176]
				lung	IR sensitivity; (-) IR-p-CHK1	[199]
**HDAC**	Class I/II	Vorinostat (SAHA)	FDA approved in 2006	multiple	IR sensitivity; (-) HR & NHEJ proteins, i.e., Rad50, Ku70; (+) γH2AX IRIF; (-) IR-induced HR & NHEJ proteins, i.e., Rad51, Ku80	[165–167,200,201]
	Class I	Romidepsin (FK228)	FDA approved in 2009	ovarian	(+) γH2AX/Rad51/53BP1 foci with cisplatin c.t., and in s.c.	[202]
				lung	IR sensitivity; (+) IR-γH2AX	[203]
				thyroid	(+)γH2AX/ROS; (-) Ku70/80 and Rad51; (+) γH2AX in s.c.	[168]
				renal cell	(+) γH2AX/ROS with 5-FU c.t. and in s.c.	[204]
	Class I/II	Panobinostat (LBH589)	FDA approved in 2015	lung, bladder	IR sensitivity; (+) IR-γH2AX; (-) Mre11/Nbs1/ Rad51 protein	[205,206]
				leukemia	(+) γH2AX; (-) DNA repair protein/signaling, i.e., Chk1/p-Chk1 with TOPIIi c.t.	[207,208]
**BRD**	BET family	JQ1	Preclinical	NMC, myeloma, prostate	-	[58,147,209]
				glioma	(-) IR-γH2AX	[71]
				leukemia	(+) γH2AX & 53BP1 foci	[210]
		I-BET151	Preclinical	leukemia, myeloma	-	[59,179]
		I-BET762	Clinical	myeloma	-	[177,179]
	CBP/p300	I-CBP112	Preclinical	leukemia	Dox sensitivity; (+) γH2AX foci with JQ1 c.t.	[180]

The inhibitor, clinical phase, cancer type, and effects on the DDR are provided for drug targeting of acetylation signaling factors. Abbreviations: CPT, camptothecin; c.t., co-treatment; Dox, doxorubicin; HU, hydroxyurea; IR, ionizing radiation; IRIF, ionizing radiation induced foci; PARPi, PARP inhibitor; ROS, reactive oxygen species; s.c., subcutaneous xenograft tumor model; TOPIIi, Topoisomerase II inhibitor; 5-FU, 5-fluorouracil. (-) indicates decreased levels; (+) indicates increased levels.

Small molecule inhibitors targeting the BRD have drawn significant attention for new classes of anticancer drugs. For example, JQ1, a potent BRD4 inhibitor, has displayed anti-tumor activities in preclinical studies [58]. In addition to JQ1, a variety of other BET family BRD inhibitors, including I-BET151 [59] and I-BET762 [177], show therapeutic promise in hematological malignancies [178]. These inhibitors have been shown to inhibit BRD4 chromatin interactions, thereby blocking MYC-mediated tumor growth and survival [59,147,179]. Reduced BRD4 function by JQ1 also alters the cellular response to ionizing radiation [71], indicating that BRD4 inhibition also impacts the DDR. A p300/CBP BRD inhibitor I-CBP112 showed enhanced topoisomerase inhibitor doxorubicin induced cytotoxicity effect in leukemic cell lines [180]. I-CBP112 also displayed a synergistic cytotoxic effect with JQ1, as more γH2AX foci were observed in co-treated cells (Table 2). These results suggest that CBP/p300 and BRD4 may function in different DDR pathways, thus providing selectivity for these inhibitors in either CBP/p300 mutant cells with BRD4 inhibitors or vice versa. These studies emphasize the promise of drugging acetylation signaling in cancer. As numerous BRD proteins are involved in the DDR, it will be vital to understand whether these BRD inhibitors impact DNA damage signaling/repair and whether these effects are advantageous or inhibitory towards the use of these compounds at therapeutic agents in cancer (Fig 3).

## Summary

Mounting evidence highlights the crucial function of acetylation signaling in regulating the DDR and maintaining genome integrity. Acetylation reader proteins, including BRD proteins, are vital effector proteins for acetylated lysines that recognize and read these signals to orchestrate the DDR. Understanding the mechanisms by which acetylation reader proteins promote chromatin-based responses to DNA damage can provide critical insights into understanding how genome–epigenome maintenance is achieved. Genome instability is common in cancer as is defective acetylation signaling pathways. Whether altered acetylation signaling is causal or merely correlative for genome instability in cancer remains an important question. How acetylation signaling impacts cancer epigenetics and its contributions to the DDR and cancer treatments warrants further investigations. Given the rapid development of epigenetic drugs targeting acetylation signaling, including BRD inhibitors, it is critical to further decipher how inhibitors of acetylation signaling affect the DDR and whether this information can be leveraged to improve the use of these drugs in cancer treatment. We envision that a deeper understanding of how acetylation signaling is involved in the DDR and in cancer will help develop targeted therapies using epigenetic drugs either alone or in combination with DNA damaging agents to improve cancer treatments.
